# A Comprehensive Approach to PROMs in Elective Orthopedic Surgery: Comparing Effect Sizes across Patient Subgroups

**DOI:** 10.3390/jcm13113073

**Published:** 2024-05-24

**Authors:** Ville Äärimaa, Karita Kohtala, Ida Rantalaiho, Elina Ekman, Keijo Mäkelä, Hanna-Stiina Taskinen, Anssi Ryösä, Joel Kostensalo, Saara Meronen, Inari Laaksonen

**Affiliations:** 1 Department of Orthopedics and Traumatology, Turku University Hospital, Luolavuorenkatu 2, 20720 Turku, Finland; ville.aarimaa@tyks.fi (V.Ä.); ida.rantalaiho@tyks.fi (I.R.); elina.ekman@tyks.fi (E.E.); keijo.makela@tyks.fi (K.M.); hanna-stiina.taskinen@tyks.fi (H.-S.T.); anssi.ryosa@tyks.fi (A.R.); saara.meronen@tyks.fi (S.M.); inari.laaksonen@tyks.fi (I.L.); 2 The Faculty of Medicine, University of Turku, Kiinamyllynkatu 4-8, 20521 Turku, Finland; 3 Natural Resources Institute Finland, Yliopistokatu 6B, 80100 Joensuu, Finland; joel.kostensalo@luke.fi

**Keywords:** treatment effect, effect size, patient-reported outcome measure (PROM), musculoskeletal disease, registry

## Abstract

**Background**: There is limited knowledge regarding the comparative patient-reported outcomes (PROMs) and effect sizes (ESs) across orthopedic elective surgery. **Methods**: All patient data between January 2020 and December 2022 were collected, and treatment outcomes assessed as a PROM difference between baseline and one-year follow-up. The cohort was divided into subgroups (hand, elbow, shoulder, spine, hip, knee, and foot/ankle). The PROM ESs were calculated for each patient separately, and patients with ES > 0.5 were considered responders. **Results**: In total, 7695 patients were operated on. The mean ES across all patient groups was 1.81 (SD 1.41), and the largest ES was observed in shoulder patients and the smallest in hand patients. Overall, shoulder, hip, and knee patients had a larger ES compared to hand, spine, and foot/ankle patients (*p* < 0.0001). The proportion of positive responders ranged between 91–94% in the knee, shoulder, and hip, and 69–70% in the hand, spine, and foot/ankle subgroups. **Conclusions**: The ESs are generally high throughout elective orthopedic surgery. However, based on our institutional observations, shoulder, hip, and knee patients experience larger treatment effects compared to hand, spine, and foot/ankle patients, among whom there are also more non-responders. The expected treatment outcomes should be clearly communicated to patients when considering elective surgery. Because of the study limitations, the results should be approached with some caution.

## 1. Introduction

Patient-reported outcome measures (PROMs) have become indispensable tools for assessing patient health, tracking changes, and evaluating treatment outcomes, particularly in non-life-threatening conditions, such as degenerative musculoskeletal diseases [1]. Disease-specific PROMs have been purposefully designed to capture even minute symptoms and changes in specific conditions, whereas generic PROMs may show no sensitivity [2,3]. Therefore, a clinically relevant change in a validated disease-specific PROM should provide the most sensitive and responsive indication of a treatment effect and benefit detectable by patients [4]. 

A multitude of musculoskeletal conditions, ranging from head to toe, are treated with continually evolving surgical options that yield diverse outcomes. For example, whereas total hip arthroplasty is advocated as one of the most effective surgical procedures [5], other operations, such as partial degenerative meniscectomies in the knees and acromioplasties in the shoulders, have been reported to provide minimal, if any, improvement in a patient’s health stage [6,7,8]. Furthermore, there are several papers on the differences in outcome between surgical techniques and factors affecting an end-result in specific musculoskeletal conditions [9,10]. 

Despite the growing number of reports on PROM outcomes in selective procedures and patient series, there is little knowledge regarding the comparative outcomes and effect sizes across the anatomic field of orthopedic elective surgery. Systematic real-world outcome data would facilitate not only the scrutiny of surgical practices but also the transparent comparisons of treatment outcomes, further research, and enhancements in standards of care [11]. Most importantly, it is in the interest of every patient to be informed of an expected treatment outcome before shared decision-making on elective operative treatment [1,12]. 

The purpose of this study was to examine and report the outcomes and effect sizes of disease-specific PROMs in elective orthopedic surgery across anatomic patient groups within a comprehensive institutional PROM registry. Our specific objective was to determine whether there is evidence indicating variations in PROM effect sizes among different patient groups. 

## 2. Materials and Methods

An institution-based anatomically divided systematic PROM registry for patients undergoing elective surgery for a musculoskeletal condition was established in 2019 at Turku University Hospital (Finland), with a catchment area of approximately 500,000 people. The registry comprises anatomic subregistries by the subspecialty of a patient’s musculoskeletal condition (i.e., hand, elbow, shoulder, spine, hip, knee, and foot/ankle). Institutionally chosen disease-specific PROM outcome instruments were used in subregistries (see Table 1). The scores were gathered preoperatively at baseline and one year postoperatively using an electronic application (Omavointi, BCB Medical, Turku, Finland). For this study, all PROM data were scaled to range from 0 to 100 and harmonized in such a way that a positive change in a score represented an improvement in a patient’s perceived quality of life. Patient history data were gathered regarding age, sex, American Society of Anesthesiology (ASA) score, and body mass index (BMI). Surgery-related data on the main diagnosis code (according to the International Classification of Diseases (ICD) 10 classification), and the main operation (according to the Classification of Surgical Procedures Version 1.14 by the Nordic Medico-Statistical Committee (NOMESCO)) were also gathered. 

All consecutive patient data between 1 January 2020 and 31 December 2022 were collected and the treatment outcome within each subregistry assessed by analyzing the difference between baseline and one-year follow-up PROM values. Patients with musculoskeletal problems due to neoplastic disease or trauma (International Classification of Diseases (ICD-10) codes from C, D, and S categories) were excluded from this study. Thus, the study included only musculoskeletal degenerative diseases that were electively operatively treated.

To compare the results between the subgroups, we adopted a unified measure of surgical outcome, which we refer to hereafter as effect size (ES). This measure can be understood as a standardized outcome measure. The ES corresponding to observation *i* is given here by:

$$ES_{i}=\frac{PROM_{12\;months,\;i}-PROM_{preop,\;i}}{SD(PROM_{preop})}$$

where PROM_12 *months*,*i*_ is the PROM response 12 months after the operation for patient *i*, PROM*_preop_*_,*i*_ is the preoperative PROM score of the patient, and SD(PROM*_preop_*) is the standard deviation of all the PROM scores in the registry. The patients with ES*_i_* > 0.5 are referred to as responders, i.e., individuals for whom a surgical treatment led to an improvement over the distribution-based estimate of minimally clinically important difference (MCID) [21]. Statistical differences in the fractions of positive responders between groups were tested using Fisher’s exact test.

The differences between subgroups were analyzed using a linear model:

$$ES_{i}=\beta_{0}+\beta_{sex}{Sex}_{i}+\beta_{BMI}BMI_{i}+\sum_{a=2}^{4}\beta_{ASA,a}\;ASA_{a,i}+\sum_{r}\beta_{reg,r}Reg_{r,i}+\epsilon_{i}$$

where the ES*_i_* is the effect size, i.e., the outcome of the treatment *i*, Sex*_i_* = 1 if the patient is female, BMI*_i_* is the body mass index of the patient in kg m^2^, ASA*_a_*_,*i*_ is a dummy variable with value 1 if patient *i* belongs to the ASA class *a*, and Reg*_r_*_,*i*_ is a dummy variable which has the value 1 if the observation *i* is from registry *r*, while *β*s are the regression coefficients to be fitted and *ϵ_i_* is an independent and normally distributed error term. Alternative models including age as a variable or ones where one of the covariates (sex, BMI, or ASA class) were left out were explored; however, based on the Akaike information criterion (AIC), the adopted model fit the data better than the other models.

It should be noted here that such selection may affect the interpretation of the *p*-values for these covariates, but these were not of interest in this work, and are treated as nuisance variables necessary for ensuring the comparability of the register ES. Comparisons between the groups were calculated as contrasts of the linear model. All statistical analyses were carried out using the statistical software R version 4.3.2 [22].

## 3. Results

In total, 74 surgeons (orthopedic specialists and residents under specialist supervision) conducted operations on 7695 patients by December 2021. By December 2022, complete one-year follow-up data from 4039 patients were available for analysis. Twenty-six specialists, all of whom had performed at least 50 surgeries, accounted for 74% of the operations. The patient demographics of the subgroups are presented in Table 2. Due to the limited number (*n* = 12) of electively operated patients with elbow disorders, they were excluded from the analyses. There were 2377 (59%) female and 1662 (41%) male patients with a mean age of 63 years (SD 14).

**Figure 1:**
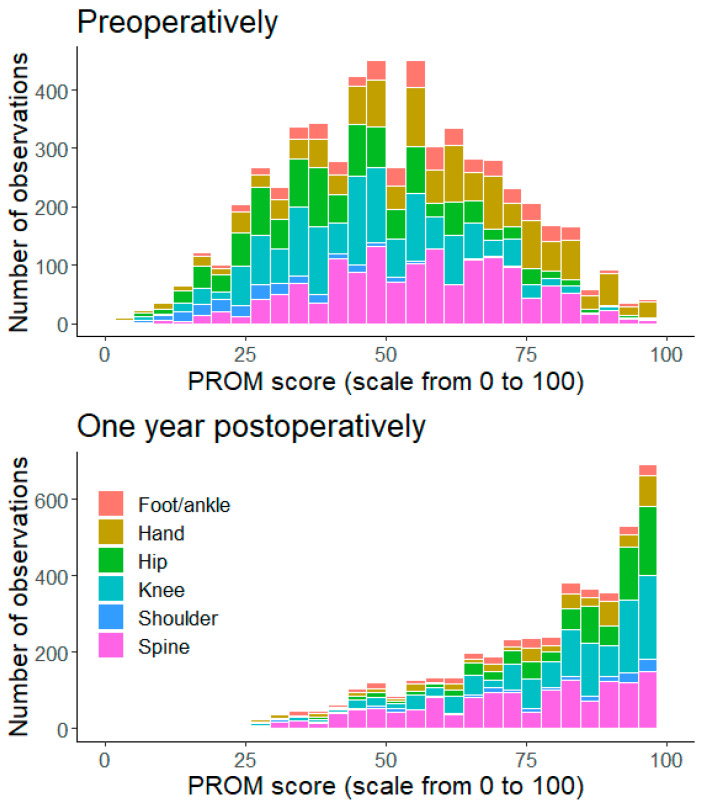
PROM measurements for all subgroups included in this study before an operation (baseline) and at the one-year follow-up mark.

The most common diagnoses and operations within each anatomic subgroup are presented in Table 3. There were 0.1% and 11.8% PROM scores hitting the floor and ceiling, respectively. The ceiling effect at the one-year follow-up was most notable in hip patients, where 24% of the patients reported a PROM measurement of 100 (see Figure 1). The mean ES was 1.81 (SD 1.41), with statistically significant differences between the subgroups, as shown in Table 4. The most substantial ES was observed among shoulder patients, while the smallest ES was observed among hand patients.

**Figure 2:**
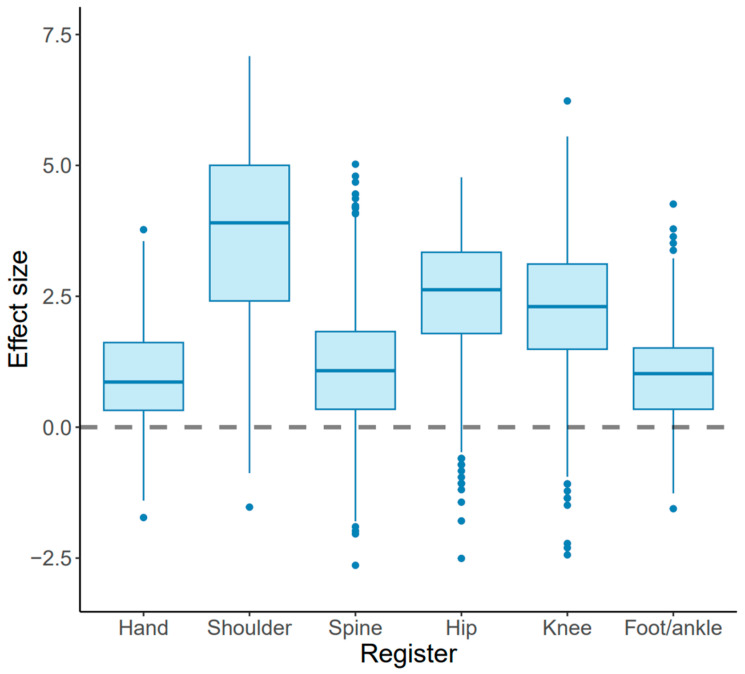
Surgical outcomes were measured as ES for the subgroups included in this study.

In general, patients undergoing shoulder, hip, or knee procedures, with ES of 3.64, 2.50 and 2.26, respectively, experienced more significant treatment effects compared to those in the hand, spine, and foot/ankle subgroups (*p* < 0.0001) (see Figure 2). The distribution-based estimates of MCID values together with the percentage of responders (positive and negative) in each patient subgroup are presented in Table 5. The percentage of patients who responded positively differed between subgroups, with the knee, shoulder, and hip subgroups showing positive responder rates ranging from 91–94%. In contrast, the hand, spine, and foot/ankle subgroups had positive responder rates of 69–70%.

Based on a post hoc power analysis, the power to detect ES differences of 0.5 with α = 0.05 was 90.2% for the shoulder and foot/ankle comparison and higher for the other comparisons. The comparisons that did not include the shoulder registry had a statistical power greater than 99%. The lower power for the shoulder comparisons reflects the smaller number of observations (N = 160) compared to the other registries (N = 304–1217).

## 4. Discussion

The primary finding of this study reveals a substantial and statistically significant difference in treatment ES based on disease-specific PROMs among anatomic patient groups undergoing elective orthopedic surgery. The biggest ES were detected among the shoulder patients. Accordingly, the number of responders and non-responders between patient subgroups was also significantly different. The lowest numbers of responders were in the hand, spine, and foot/ankle subgroups. Nonetheless, the mean ES were higher than the estimated clinically significant levels in all patient groups. 

Substantial heterogeneity has been observed in the effects of surgical treatment in general [23]. In Sung et al.’s study, the average ES in orthopedic trials was reported to be 1.7 [24], which is essentially in accordance with our findings. A generally accepted limit for a large ES in clinical medicine is 0.8 [25]. Within our study, we observed that the average ES among the shoulder, hip, and knee patients was notably higher when compared to patients with hand, spine, and foot/ankle conditions.

We are not aware of similar reports on the comparative PROM ES of orthopedic procedures. However, Gates et al. reported a high utility and gain in quality-adjusted life years in hip and knee arthroplasty compared to other common surgical procedures [26]. In line with this, the majority of our hip and knee patients, along with shoulder patients, underwent total joint arthroplasty procedures. Hip and knee arthroplasties are renowned for their high patient satisfaction [1], with hip arthroplasty earning the distinction of being named “the operation of the century” [27].

Although shoulder arthroplasties are less frequent, previous validation studies have reported similarly high ESs [28,29]. Moreover, extensive research has been conducted to study the effectiveness and indications for arthroscopic procedures in shoulder and knee patients. These efforts have resulted in notable shifts in treatment paradigms and a reduction in the number of ineffective arthroscopic procedures [6,7,30,31,32]. It is noteworthy that, in our study, there was a greater variability in the types of operations performed in other patient subgroups compared to shoulder, hip, and knee patients.

Musculoskeletal conditions encompass 16% of the global disease burden, rendering their impact on public health greater than that of any other disease group [33]. Therefore, the open dissemination of health care outcomes and the value of elective orthopedic surgery may be regarded as the responsibility of all care providers [34].

PROMs serve as assessments of fundamental aspects of health and can also be viewed as quality indicators for healthcare, given their ability to capture patient-perceived adverse events and care-related complications. Nonetheless, one must bear in mind that changes in subjective health after a surgical intervention comprise both specific and nonspecific effects [35], and sometimes the latter might be of greater significance. Therefore, the ESs in our study do not represent differences in the effect of surgical intervention per se but rather differences in the overall perceptions of our treated patients. Jayakumar et al. reported that the mental and social health of a trauma patient had a significant impact on the pain experienced by the patient and the patient’s general ability to function [36]. Psychological factors also play an important role in elective orthopedic treatment outcomes [35,37]. 

We employed 10 different PROMs selected based on expert opinions from our specialists. It is important to acknowledge that disease-specific PROM scoring may not have been optimally suited for all patient subgroups despite a shared denominator of degenerative joint conditions. Although the transformation to effect size makes the PROMs more comparable, more nuanced differences in the distributions of PROM scores, such as ceiling effects, detected in the hip group in our study, cannot be resolved [38]. Due to the different psychometric properties of PROMs, this kind of simple effect-size comparison may be criticized [39]. The concepts of the responder and MCID are also ambiguous, in addition to being context- and patient-group-dependent [40].

There are over 40 validated and, to some extent, competing disease-specific PROMs for musculoskeletal conditions [2]. Despite the best efforts of, for example, the International Consortium of Health Outcome Measures (ICHOM) and consensus-based standards for the selection of health measurement instruments (COSMINs), there is a lack of uniform inclusive commitment [4,41]. Furthermore, PROM validation should proposedly be an iterative cross-cultural process instead of a static designation that a PROM is valid [3]. Only seven of our ten PROM questionnaires have been properly cross-culturally validated in Finnish thus far [42,43,44,45,46,47,48], and, in the remaining three PROMs, we used an institutionally translated version, yet without published data on psychometrics.

The response burden also plays a significant role in registry coverage, and this may influence the results [49,50,51]. In our study, the response coverage in some subregistries was below 60%, potentially due to the lengthy questionnaires. Consequently, there is a need for the further development of short and valid disease-specific PROMs [49,50]. It would also be good to assess whether answering the survey could be made easier for the patient via technical means. Individualized shortened questionnaires (e.g., the National Institute of Health’s Patient-reported Outcomes Measurement Information System (PROMIS)) that adapt outcome scores are increasingly replacing the first-generation disease-specific PROMs used in our study. However, their sensitivity may be poor as they are more generic in nature [52].

### Limitations and Strengths

We acknowledge that, in addition to the potential criticism related to PROM selection, our study has weaknesses. First, although we included only degenerative diagnosis, there may have been acute on chronic cases, degenerative conditions with a purely traumatic etiology, or revision cases with failed previous surgery in all subgroups. Second, lumping anatomic subgroups without considering, for example, the pathophysiological subgroup differences in detail may be criticized. However, the purpose of this study was to report the real-world effect size of clinical practices as they are without attributing causality or implying a matter of choice. Third, despite the analyses being controlled for sex and age [53], we could not control for all potential confounders, such as comorbidities and socioeconomic factors. Fourth, we lacked a fully comparable generic PROM or a uniform global score for all patients. However, given that a disease-specific PROM provides the most sensitive tool for indicating a treatment effect for each condition, it is plausible that, with a generic PROM, the detected effect sizes would have been significantly smaller. Fifth, a large divergence in the same PROM MCID estimates between different patient populations has been reported [54], and our responder analysis was based on distribution only. 

As a strength, this was a systematic, comprehensive, tertiary, hospital-based registry study with a large number of consecutive patients. We included fully completed data only; given the number of patients, it is likely that the detected differences represent actual patient-group differences and are not dependent on individual fluctuations. The one-year follow-ups were likely sufficient for capturing the largest treatment effect [33]. Our study’s novel setup allows for a further cost–benefit analysis of the value of the provided care. Moreover, our study provides a model for everyday reporting and effect-size comparisons throughout elective surgery.

## 5. Conclusions

The observed effect sizes are generally high throughout elective orthopedic surgery for degenerative conditions. However, a substantial difference exists among various anatomic patient groups. On average, shoulder, hip, and knee patients experience larger treatment effects compared to hand, spine, and foot/ankle patients, among whom there are also more non-responders. The expected treatment outcomes should be clearly communicated to patients when considering elective surgery. 

Further research should be targeted at enhancing PROM implementation, identifying the reasons for effect-size variation, and improving treatment outcomes throughout orthopedics, and especially in patient groups with small treatment-effect sizes.

## Figures and Tables

**Table 1:** Subregistries, subgroups, and disease-specific patient-reported outcome measures (PROMs).

Patient Group	PROM	Previously Reported MCID	Reference
Hand	Disability of Arm, Shoulder and Hand (quickDASH)	15.7 ± 8.4 (7.3–24.1%)	[13]
Shoulder instability	Western Ontario Shoulder Instability (WOSI)	510.0 ± 83.4 (20.3–28.3%)	[13]
Shoulder rotator cuff	Western Ontario Rotator Cuff (WORC)	343.6 ± 94.3 (11.9–20.9%)	[13]
Shoulder arthroplasty	Western Ontario Osteoarthritis of Shoulder (WOOS)	12.3 (12.3%)	[14]
Cervical spine	Neck Disability Index (NDI)	8.5 (17%)	[15]
Lumbar spine	Oswestry Disability Index (ODI)	7.5 (7.5%)	[16]
Hip	Oxford Hip Score (OHS)	5.2 (10.8%)	[17]
Hip arthroplasty	Oxford Hip Score (OHS)	5.2 (10.8%)	[17]
Knee	Knee Injury and Osteoarthritis Outcome Score (KOOS)		
	Pain	7.9 (7.9%)	[18]
	Symptoms	1.2 (1.2%)	
	Activities of Daily Living (ADL)	8.1 (8.1%)	
	Sport and Recreational Function (Sport/Rec)	21.7 (21.7%)	
	Knee-Related Quality of Life (QOL)	27.3 (27.3%)	
Knee arthroplasty	Oxford Knee Score (OKS)	4.7–10 (9.8–20.8%)	[19]
Foot/Ankle	Foot and Ankle Outcome Score (FAOS)	14 (20.1%)	[20]

**Table 2:** Demographics in patient subgroups.

	Age: Mean (SD)	Female (%)	BMI: Mean (SD)	ASA ≥ 3	Surgeons	Response Coverage	N
Hand (quickDASH)	56 (13)	64	29.6 (6.0)	19%	12	24%	403
Shoulder (WOSI, WORC, WOOS)	62 (15)	53	28.8 (4.9)	34%	13	57%	160
Spine (NDI, ODI)	60 (15)	51	28.2 (4.8)	38%	36	57%	1217
Hip (OHS)	67 (12)	60	28.4 (4.6)	38%	21	67%	843
Knee (OKS, KOOS)	68 (11)	62	29.6 (4.4)	41%	25	69%	1112
Foot/Ankle (FAOS)	56 (14)	71	28.2 (5.2)	21%	15	42%	304
ALL	63 (14)	59%	28.9 (4.9)	36%	74	52%	4039

**Table 3:** The most common diagnoses and operations in patient subgroups. Note that both numbers refer to the entire registry; thus, the operation percentages do not refer only to the diagnosis in the same row of the table.

	Diagnosis		Operation	
Hand (quickDASH)	G56.0	26%	ACC51	22%
	M72.0	8%	NDM10	8%
	M65.3	5%	NDM40	5%
Shoulder (WOSI, WORC, WOOS)	M19.0	54%	NBB20	56%
	M75.1	18%	NBL05	13%
	M24.4	9%	NBL22	7%
Spine (NDI, ODI)	M48.0	35%	ABC16	18%
	G55.1*M51.1	11%	ABC56	17%
	M51.1	10%	ABC36	15%
Hip (OHS)	M16.1	49%	NFB40	64%
	M16.0	35%	NFB30	26%
	T84.0	3%	NFC20	3%
Knee (OKS, KOOS)	M17.1	47%	NGB20	85%
	M17.0	40%	NGB10	4%
	T84.0	2%	NGB40	2%
Foot/Ankle (FAOS)	M20.1	24%	NHG80	20%
	M19.0	12%	NHG26	13%
	M20.2	8%	NHK30	10%

**Table 4:** Mean ES, their 95% confidence intervals, and results from comparisons of ES. The superscript letters for the mean ES denote statistical significance. Groups with mutually exclusive letters differ in a statistically significant way (i.e., hand and foot/ankle do not differ in a statistically significant way, while all other register pairs do differ at the *p* < 0.05 level).

	Mean Effect Size	95%-C.I.
Hand (quickDASH)	0.93 ^A^	[0.84, 1.03]
Shoulder (WOSI, WORC, WOOS)	3.64 ^B^	[3.36, 3.92]
Spine (NDI, ODI)	1.14 ^C^	[1.07, 1.20]
Hip (OHS)	2.50 ^D^	[2.42, 2.58]
Knee (KOOS, OKS)	2.26 ^E^	[2.18, 2.33]
Foot/Ankle (FAOS)	1.01 ^A^	[0.90, 1.12]

**Table 5:** The percentage of patients with a clinically significant change in the PROM score (ES > 0.5SD or ES < −0.5SD). The superscript letters in the last two columns denote statistically significant differences between the groups.

	MCID Estimate 0.5 SD	Clinically Significant Deterioration	Clinically Significant Improvement
Hand (quickDASH)	10.5	6% ^A^	70% ^A^
Shoulder (WOSI, WORC, WOOS)	6.8, 6.0, 7.1	2% ^BC^	93% ^BC^
Spine (NDI, ODI)	8.3, 8.8	6% ^AB^	69% ^A^
Hip (OHS)	8.7	1% ^C^	94% ^B^
Knee (KOOS, OKS)	10.3, 7.7	2% ^C^	91% ^C^
Foot/Ankle (FAOS)	9.3	6% ^AB^	70% ^A^

## Data Availability

The datasets presented in this article are not readily available because hospital data contains patient identification data, and GDPR prevents us from sharing individual raw data. Requests to access the datasets should be directed to the corresponding author.
